# Differential sensitivity and specificity of *Aedes aegypti* and *Anopheles gambiae* to adenine nucleotide phagostimulants—an all-or-none response?

**DOI:** 10.1186/s13071-024-06482-4

**Published:** 2024-11-04

**Authors:** Matthew Lukenge, Rickard Ignell, Sharon Rose Hill

**Affiliations:** Disease Vector Group, Unit of Chemical Ecology, Department of Plant Protection Biology, Swedish University of Agricultural Sciences, Alnarp, Sweden

**Keywords:** Adenosine triphosphate, Blood feeding, Mosquito, Feeding stimulants, Prediuresis

## Abstract

**Background:**

The decision to imbibe a blood meal is predominantly dependent on the sensitivity and specificity of haematophagous arthropods to blood-derived adenine nucleotides, in particular adenosine triphosphate (ATP). Despite previous efforts to identify and characterise the specificity and sensitivity to ATP and other adenine nucleotides, as well as the role of other blood-derived phagostimulants across the Culicidae, comparisons across species remain difficult.

**Methods:**

The feeding response of the yellow fever mosquito *Aedes aegypti* and the African malaria vector *Anopheles gambiae* to adenine nucleotides in the presence of a carbonate buffer was assessed using a membrane feeding assay. The proportion of mosquitoes engorged and the volume imbibed by all mosquitoes was scored visually and spectrophotometrically. In addition, the proportion of prediuresing *An. gambiae*, as well as the volume engorged and prediuresed, was examined.

**Results:**

*Aedes aegypti* was more sensitive to adenine nucleotides than *An. gambiae*, but both species maintained specificity to these phagostimulants, demonstrating a dose-dependent bimodal feeding pattern, thereby expanding our understanding of the all-or-none blood-feeding hypothesis. Feeding on the bicarbonate buffer by *An. gambiae*—but not that of *Ae. aegypti*—demonstrated a species-specific variation in how blood phagostimulants are encoded. Adenine nucleotides, with and without bovine serum albumin, were observed to dose-dependently regulate the proportion of *An. gambiae* prediuresing and the volumes prediuresed but not volumes engorged.

**Conclusions:**

Taken together, the results of this study expand our understanding of how mosquitoes differentially assess and respond to blood meal constituents, and provide a basis for further physiological and molecular studies.

**Graphical Abstract:**

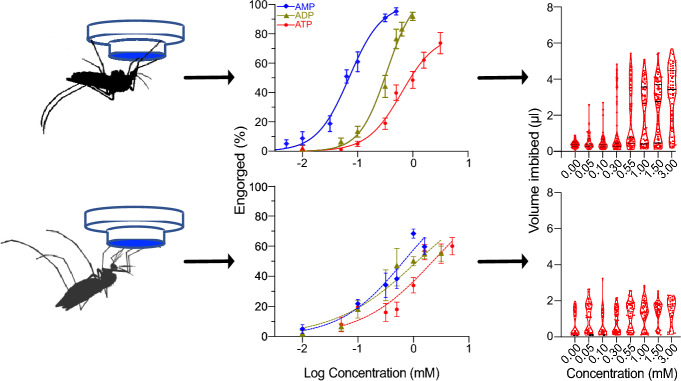

**Supplementary Information:**

The online version contains supplementary material available at 10.1186/s13071-024-06482-4.

## Background

Haematophagous arthropods have evolved independently, yet the majority detect adenine nucleotides as an indicator of an acceptable blood meal using a conserved mechanism [1]. While the majority of blood-feeding mosquitoes, such as the yellow fever mosquito *Aedes aegypti*, use adenosine triphosphate (ATP) as the major phagostimulant in blood [2–5], several others, including *Culex* and *Culiseta* species, use adenosine diphosphate (ADP) [6, 7], or are reported to be indifferent to adenine nucleotides. For example, in one study the need for ATP as a phagostimulant could not be demonstrated in select *Anopheles* species [8]. Blood- or adenine nucleotide-induced feeding in arthropods has generally been described as an all-or-none event in which partial engorgement is unusual, even when feeding on low-quality diets [9–11]. In the present study, the behavioural response of *Ae. aegypti* and the African malaria vector, *Anopheles gambiae *sensu stricto (*An. gambiae *s.s.), was directly compared to assess whether these species differentially detect and feed on the natural adenine nucleotides.

While little is known about the behavioural response of *An. gambiae* to adenine nucleotides [8, 12], the feeding response of *Ae. aegypti* is well characterised [1, 2, 4, 13, 14]. The length of the phosphate chain regulates the behavioural sensitivity to the phagostimulant, with the highest sensitivity to ATP and ranking in the order of ATP > ADP > AMP (adenosine monophosphate) > cAMP (cyclic AMP) [13]. In addition, the amino (carbon 1) and hydroxyl groups (carbons 2 and 3) of the adenine nucleotides are required in the initiation of feeding [13]. Other components of the blood meal*,* such as sodium chloride (NaCl), sodium bicarbonate (NaHCO_3_), albumin and glucose, also stimulate and induce blood-feeding by acting together through combinatorial coding, in the presence of adenine nucleotides in, for example, *Culex pipiens f. pallens* [2, 15], *Ae. aegypt*i [4, 16], or in their absence, for example, select *Anopheles* spp. [8]. Indeed, using these identified components, artificial diets for *Ae. aegypti* and *An. gambiae* have been developed and analysed [12, 13, 16]. The variation in feeding response to adenine nucleotides within the Culicidae family has the potential to shed light on the adaptations that led to the convergent evolution of the haematophagic behaviour in arthropods.

Despite previous efforts to identify and characterise the specificity and sensitivity to adenine nucleotides across the Culicidae, comparisons across species, particularly those exhibiting differential feeding associated with these ligands, remain difficult, as experimental conditions have varied between studies. For this reason, we assessed the proportion of individuals feeding and the volumes of diets imbibed by *Ae. aegypti* and *An. gambiae* s.s. in response to phagostimulation by the four naturally occurring adenine nucleotides in a dose-dependent manner, using the same behavioural assay. We discuss our findings in relation to the proposed mechanisms for the evolution of adenine nucleotide detection.

## Methods

### Mosquito rearing

*Aedes aegypti* (Rockefeller) and *An. gambiae* s.s. (G3L) mosquitoes were reared and maintained at 25 ± 2 °C and 70 ± 5% relative humidity under a 12/12-h light/dark photoperiodic regimen, as previously described [17, 18]. Briefly, eggs of the two species were separately hatched in larval trays (23.5 cm × 18 cm × 7.5 cm; ca. 300 ml water), with approximately 300 larvae per tray that were fed daily on Tetramin® fish food (Tetra, Melle, Germany). Pupae were collected into 30-ml cups (Nolato Hertila, Åstorp, Sweden) and transferred to Bugdorm cages (30 cm × 30 cm × 30 cm; MegaView Science, Taichung, Taiwan) where adults emerged. For colony maintenance, adults of both sexes were maintained with ad libitum access to 10% sucrose, and females were fed on sheep blood (Håtuna Lab, Bro, Sweden) using a membrane feeding system (Hemotek Ltd., Blackburn, UK). For the experiments, non-blood-fed females of both species, previously provided access to sucrose up to 4 days post-eclosion (dpe), were used. The sexes were not separated and the females were presumed to have mated. Thereafter, mosquitoes were starved (22 ± 2 h) with access only to water until 2 h prior to the feeding experiments [3].

### Feeding bioassay

**Fig. 1:**
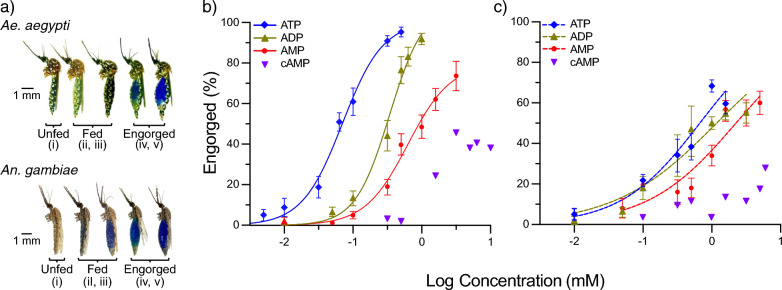
Sensitivity and specificity of *Aedes aegypti* and *Anopheles gambiae* to adenine nucleotide feeding stimulants. **a** Scale (i-v) used to score unfed to engorged individuals [34].** b**,** c** Dose-dependent response in the proportion of *Ae. aegypti* (**b**) and *An. gambiae* (**c**) mosquitoes engorged on ATP, ADP, AMP and cAMP. The buffer without nucleotides was the negative control (0 mM; not shown on the log scale). No curves were generated for cAMP since the proportion of mosquitoes engorging were below the 0.5 proportion level. Error bars indicate the standard error of the proportion. The number of replicates for each diet were *N* = 9 for* Ae. aegypti*, with each replicate consisting of 90 mosquitoes * n* = 90), and *N* = 5 (*n* = 50 mosquitoes) for *An. gambiae*. ADP, Adenosine diphosphate; AMP, adenosine monophosphate; ATP, adenosine triphosphate; cAMP, cyclic adenosine monophosphate

To establish the sensitivity and specificity of the adenine nucleotides in both species, various concentrations (0.01–15 mM) were prepared by serial dilution from stock solutions in xylene cyanol FF dye (1 mg ml^−1^; Merck, Darmstadt, Germany). An initial panel of concentrations was chosen based on those used previously by Galun for *Ae. aegypti* [3, 4, 13] and those used previously for *Anopheles stephensi*, *Anopheles dirus* and *Anopheles freeborni* [8] for *An. gambiae*, with additional concentrations being added to clarify the half-maximal effective concentration (EC_50_) inducing 50% feeding, where possible. To prepare the stock solutions for the feeding assays, ATP (2 mM; CAS no. 34369-07-8; Merck), ADP sodium salt (10 mM; CAS no. 20398-34-9; Merck), AMP disodium salt (20 mM; CAS no. 4578-31-8; Merck) or cAMP (20 mM; CAS no. 60–92-4; Merck) were dissolved in bicarbonate-buffered saline (150 mM NaCl, 10 mM NaHCO_3_, pH 7.4 ± 0.07) and stored at − 20 °C. Using the membrane feeding system (Hemotek Ltd.) and collagen membrane-covered reservoirs (0.3 ml) filled with 200 μl of each adenine nucleotide concentration or buffer alone, mosquitoes were provided access to heated diets at 37 °C for 30 min. Prior to exposure, 10 female mosquitoes (5 dpe) were gently aspirated into tall Petri dishes (diameter 12 cm, height 6 cm height; Semadeni, Ostermundigen, Switzerland) covered by a plastic lid with a window of fine mesh. For each concentration of each adenine nucleotide, a total of 90 mosquitoes (*n* = 90) from nine separate cohorts of each species (*N* = 9 replicates) were bio-assayed. Since *Ae. aegypti* and *An. gambiae* are diurnal and nocturnal feeders, respectively, the assays were performed during their peak biting periods, i.e. the photophase (Zeitgeber time [ZT] 8–10) and scotophase (ZT 13–15), respectively [19–21]. Mosquitoes with dye visible in their extended abdomen were scored as engorged following the scoring scales from which the proportion of engorged mosquitoes was estimated (Fig. 1a). Approximately 20% of the *An. gambiae* engorged on the buffer alone; therefore, the proportion of engorged mosquitoes responding to adenine nucleotides was corrected for by deducting the buffer effect. All of the mosquitoes exposed to the diet were anaesthetised at − 20 °C, individually placed in 1.5-ml microfuge tubes and stored at − 20 °C until analysis by spectrophotometry.

### Volumetric estimation of imbibed diet

To establish whether feeding is an all-or-none response, the volume of each diet imbibed was examined using microplate spectrophotometers (λ620 nm; SPECTROStar® Nano, BMG Lab Tech, Ortenberg, Germany; Multiscan™ FC, Fisher Scientific, Stockholm, Sweden) according to Dawit et al. [22]. The dye content from the mosquito gut was released into 240 μl of Milli-Q water using a disposable pestle and a cordless motor (VWR International, Stockholm, Sweden), and then 200 μl of the supernatant was transferred to a clear polystyrene flat-bottomed 96-well spectrophotometer microplate (Fisher Scientific). A standard curve to determine the volume of dye imbibed by each mosquito was prepared by serial dilution of xylene cyanol FF in the supernatant extracted from the equivalent of an unfed mosquito ground in Milli-Q water (200 µl), to account for any background absorbance attributed to the mosquito. To determine the volumetric resolution of the spectrophotometer for xylene cyanol FF, we analysed the variation between the standard curve replicates and doses, and identified an optical density of 0.052 (1 mg) as the threshold for reliable detection. Any readings below this value were considered to be unfed mosquitoes. The volumes imbibed were examined by spectrophotometry among the individuals that were visually scored as engorged or fed, categorised as and referred to as engorgers and tasters, respectively.

### Proportion prediuresing, volumes imbibed and prediuresed in *An. gambiae*

Prediuresis, the process of fluid being excreted from the rectum of an actively feeding mosquito, was observed in *An. gambiae*. While small droplets of prediuretic fluids have been reported in *Ae. aegypti* [23, 24], this was not observed in the present study and previous studies [25–27]. The proportion of prediuresing *An. gambiae* and the volume imbibed by prediuresing *An. gambiae*, as well as their average prediuresed volume, were measured by modifying the membrane feeding protocol described in the section Feeding bioassay. In brief, pools of ten 5-dpe mosquitoes were gently aspirated into conical centrifuge tubes (50 ml; VWR International) that were then covered with a top fine mesh and tightened with rubber bands, and the mosquitoes then provided with diets containing 0.01 mM, 0.55 mM and 1 mM of ATP, ADP and AMP, as well as the buffer control; each diet was tested in seven to nine replicates. Reservoirs containing 200 μl of each ligand meal were placed on top of the fine mesh, exposing the mosquitoes for 30 min; the proportion of individuals that prediuresed and engorged was recorded. Each engorged individual was then placed in a 1.5-ml microfuge tube and stored at − 20 °C until analysis by spectrophotometry to determine the volumes imbibed, as described in section Volumetric estimation of imbibed diet. Distilled water (240 μl) was used to dilute and repeatedly wash the prediuresed fluid from the Falcon tube walls. This was followed by a quick spin (4460 g) in an ultracentrifuge (Sorvall RC-5B; Dupont instruments, Stockholm, Sweden). To estimate the average volume prediuresed by each individual, we first determined the total volume prediuresed in each pool of prediuresing females using spectrophotometry (as described) and then divided the total volume by the number of prediuresed individuals. To account for the possible effect of a protein source in an artificial blood meal on prediuresis, bovine serum albumin (BSA 5%; CAS no. 9048-46-8; Merck) was added to the ATP diet, following which the proportion of *An. gambiae* engorged and prediuresed, as well as the volumes that were engorged and prediuresed across concentrations, was examined.

### Statistical analysis

A non-linear regression curve fit model using a least-square regression fitting method with the bottom and top constrained to 0% and 100% (minimum and maximum proportions) was used to test the sensitivity and specificity between and within species in response to the adenine nucleotides, with respect to the proportion of individuals feeding, by comparing whether the best-fit values of unshared parameters differed between the different datasets. The parameters evaluated included the exposure concentration per dose at 50% engorgement (EC_50_) and the slope of the curves (Hill Slope). The proportion of engorged mosquitoes and log-concentrations were considered to be the dependent and independent variables, respectively. To test for the differences in the volumes imbibed within and between ligands of each individual species, we used an ordinary two-way analysis of variance (ANOVA) followed by a Tukey’s post-hoc multiple comparisons test to compare volumes imbibed within the dynamic range of each ligand. Moreover, to examine the differences in the proportion of prediuresing *An. gambiae,* the volumes imbibed and the average volumes prediuresed (the response variable was normalised with square root transformation), we used a two-way ANOVA. Upon the addition of BSA in the ATP diets, the proportion of engorged and prediuresed individuals, as well as the engorged and the average prediuresed volumes, across concentrations were tested using a one-way ANOVA followed by a Tukey’s post-hoc analysis. The values for the average volumes prediuresed were normalised with a square root transformation. Since mosquitoes did not exhibit a clear and consistent dose-dependent feeding response to cAMP, this tastant was not included in the volumetric analyses. All statistical analyses were performed using GraphPad Prism (version 10.0.0; GraphPad Software, San Diego, CA, USA; www.graphpad.com).

## Results

### Adenine nucleotides regulate feeding in a dose- and species-dependent manner

To determine the sensitivity and specificity of both *Ae. aegypti* and *An. gambiae* to the four natural adenine nucleotides, we visually scored the proportion of mosquitoes that were engorged (Fig. 1a). The feeding response of both species was dose-dependent, and those adenine nucleotides with the higher number of phosphates were ranked as the more potent phagostimulants: ATP > ADP > AMP > cAMP. *Aedes aegypti* was more sensitive to these ligands than* An. gambiae* (*F*_(5, 288)=_ 108.4; *P* < 0.0001; (Fig. 1b, c; Tables 1, 2, 3, 4). While both species were sensitive to cAMP, this ligand did not elicit engorgement in > 50% of the mosquitoes tested and, therefore, was not included in further analyses. In *Ae. aegypti*, the dynamic window of response to ATP and ADP did not differ but they were significantly different from that of AMP (Fig. 1b; Table 2). In contrast, the dynamic window (slopes) of response to the adenine nucleotides was similar across all three ligands in *An. gambiae* (Fig. 1c; Table 3). While *Ae. aegypti* did not engorge on the saline buffer control in the absence of adenine nucleotides, a proportion of *An. gambiae* fed on the buffer alone (20% ± 0.10%).

**Table 1:** Sensitivity and dynamic range of *Aedes aegypti* and *Anopheles gambiae* to adenine nucleotides

Parameter	*Aedes aegypti*			*Anopheles gambiae*		
	ATP	ADP	AMP	ATP	ADP	AMP
HillSlope	1.59 ± 0.030	2.02 ± 0.031	1.11 ± 0.040	0.75 ± 0.066	0.59 ± 0.096	0.68 ± 0.075
EC_50_	0.071 ± 0.19	0.305 ± 0.25	1.01 ± 0.12	0.67 ± 0.13	1.17 ± 0.10	2.24 ± 0.096

Values (mean ± standard error of the mean) were obtained using a non-linear regression curve fit model using a least-square regression statistical fitting method

*ADP* Adenosine diphosphate,* AMP* adenosine monophosphate,* ATP* adenosine triphosphate,* EC*_*50*_ half maximal effective concentration (M)

**Table 2:** Sensitivity and dynamic range comparisons between ligands in *Aedes aegypti*

Comparison	EC_50_		HillSlope	
	*F* (DFn, DFd)	*P* value	*F* (DFn, DFd)	*P* value
ATP vs. ADP	156.80 (1, 115)	< 0.0001	1.61 (1, 115)	0.21
ADP vs. AMP	113.60 (1, 122)	< 0.0001	16.40 (1, 122)	0.00030
ATP vs. AMP	349.60 (1, 119)	< 0.0001	7.07 (1, 119)	0.021

Values were obtained using a non-linear regression curve fit model using a least-square regression statistical fitting method

*ADP* Adenosine diphosphate,* AMP* adenosine monophosphate,* ATP* adenosine triphosphate,* DFd* degree of freedom for the denominator of the* F* ratio,* DFn* degree of freedom for the numerator of the F ratio, * EC*_*50*_ half maximal effective concentration (M)

**Table 3:** Sensitivity and dynamic range comparisons between ligands in *Anopheles gambiae*

Comparison	EC_50_		HillSlope	
	*F* (DFn, DFd)	*P* value	*F* (DFn, DFd)	*P* value
ATP vs. ADP	4.50 (1, 66)	0.040	ns	
ADP vs. AMP	4.67 (1, 73)	0.034	ns	0.58
ATP vs. AMP	20.73 (1, 65)	< 0.0001	ns	

Values were obtained using a non-linear regression curve fit model using a least-square regression statistical fitting method

*ADP* Adenosine diphosphate,* AMP* adenosine monophosphate,* ATP* adenosine triphosphate,* DFd* degree of freedom for the denominator of the* F* ratio,* DFn* degree of freedom for the numerator of the F ratio, * EC*_*50*_ half maximal effective concentration (M)

**Table 4:** Species sensitivity comparisons across ligands

Adenine nucleotide	*Ae. aegypti*	*An. gambiae*	F (DFn, DFd)	*P* value
ATP	0.071 ± 0.19	0.67 ± 0.13	84.24 (1, 94)	< 0.0001
ADP	0.305 ± 0.25	1.17 ± 0.10	45.66 (1, 96)	< 0.0001
AMP	1.01 ± 0.12	2.24 ± 0.096	23.42 (1, 98)	< 0.0001

Values are EC_50_ values and were obtained using a non-linear regression curve fit model using a least-square regression statistical fitting method

*ADP* Adenosine diphosphate,* AMP* adenosine monophosphate,* ATP* adenosine triphosphate,* DFd* degree of freedom for the denominator of the* F* ratio,* DFn* degree of freedom for the numerator of the F ratio, * EC*_*50*_ half maximal effective concentration (M)

### Phagostimulation by adenine nucleotides induced a bimodal feeding response

**Fig. 2:**
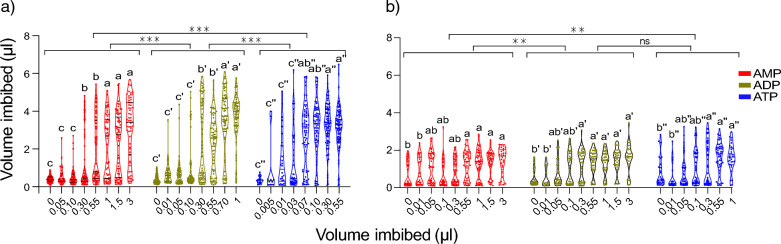
The bimodal feeding pattern on adenine nucleotides in *Aedes aegypti* (**a**) and *Anopheles gambiae* (**b**) showing a dose-dependent increase in the volumes of AMP, ADP, and ATP imbibed. Different lowercase letters above bars indicate significant differences within ligands, with the single and double primes referring specifically to ADP (') and ATP (''), respectively, as determined by pairwise post-hoc tests. Asterisks indicate a significant difference between ligands, determined by two-way analysis of variance at **P = 0.0010 and ***P < 0.001. For *Aedes aegypti*, the number of replicates for each diet was *N* = 9, with each replicate consisting of *n* = 90 *Ae. aegypti* (row effect: *F*_(11, 1184)_ = 38.90; *P* < 0.001; column effect: *F*_(2, 1184)_ = 142.1, *P* < 0.001); for *An. gambiae*, the number of replicates for each diet was *N* = 5, with each replicate consisting of *n* = 50 *An. gambiae* (row effect: *F*_(7, 735)_ = 7.758; *P* < 0.001; column effect: *F*_(2, 735)_ = 8.223, *P* < 0.001). A main effects model was used due to the different dynamic range of concentrations tested for each ligand. ADP, Adenosine diphosphate; AMP, adenosine monophosphate; ATP, adenosine triphosphate; ns, non-significant

To further characterise how mosquitoes respond to adenine nucleotides, we performed volumetric estimations of the diet imbibed using spectrophotometry*.* In *Ae. aegypti*, while the volume imbibed within the dynamic range during feeding on adenine nucleotides was dose-dependent, the distribution of these volumes was bimodal in nature, with mosquitoes displaying a ranked sensitivity to the length of the phosphate chains of the adenine nucleotides, i.e. ATP > ADP > AMP (Fig. 2a). In *An. gambiae,* the imbibed volumes in response to AMP were significantly different from those of the other nucleotides, whereas the response to ADP and ATP did not differ (i.e. ATP = ADP > AMP; Fig. 2b). The bimodal distributions of volumes, regardless of species or ligand, described a two-phase feeding response with tasting and engorging categories (Fig. 2a, b). In *Ae. aegypti*, the average volume within tasters alone (AMP 0.48 ± 0.021 μl; ADP 0.55 ± 0.020 μl; ATP 0.44 ± 0.033 μl) and engorgers alone (AMP 3.23 ± 0.14 μl; ADP 3.80 ± 0.12 μl; ATP 3.65 ± 0.051 μl) was not significantly different within the respective categories (*P* > 0.05; Additional file 1: Figure S1a). Moreover, a similar bimodal distribution in *Ae. aegypti* was observed in *An. gambiae*, i.e. tasters (AMP 0.25 ± 0.020 μl; ADP 0.29 ± 0.013 μl; ATP 0.31 ± 0.022 μl) and engorgers (AMP 1.66 ± 0.030 μl; ADP 1.65 ± 0.039 μl; ATP 1.84 ± 0.058 μl) (Additional file 1: Figure S1b) in which no significant difference was observed within the categories.

### Prediuresis did not affect the volume engorged in *An. gambiae*

**Fig. 3:**
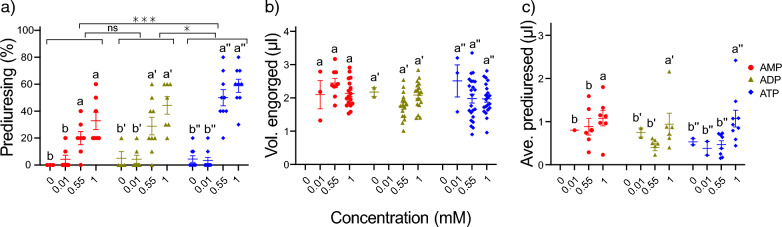
The effect of adenine nucleotides on prediuresis in *Anopheles gambiae*. **a** The percentage of prediuresing individuals, **b** the volume engorged among prediuresing individuals, and **c** the average volume prediuresed (**c**). Lowercase letters indicate the levels of significance within the ligands, with the single and double primes referring specifically to ADP (') and ATP (″) ligands, respectively. The error bars indicate the standard error of the proportion and the volumes engorged and prediuresed. (*N* = 7–9 replicates, *n* = 70–90* An. gambiae* per replicate). Different lowercase letters indicate a significant difference as determined by pairwise post-hoc tests. Asterisks indicate a significant difference between ligands determined by a two-way analysis of variance at *P < 0.05 and ***P < 0.001. ADP, Adenosine diphosphate; AMP, adenosine monophosphate; ATP, adenosine triphosphate; ns, non-significant

The potential effect of prediuresis on the volume imbibed was examined using a modified membrane feeding assay. With few exceptions, the engorged individuals also prediuresed; however, variable rates of prediuresis were observed. A significant dose-dependency was demonstrated in the proportion of prediuresing individuals (Fig. 3a), which was similar to the proportions engorged (Additional file 1: Figure S1c), and was observed across the ligands, with ATP eliciting the highest proportion of prediuresing individuals (Fig. 3a). While the engorgement threshold in response to ATP increased in the presence of BSA, the addition of BSA to this diet neither altered the proportion nor the average volume prediuresed (Additional file 2: Figure S2). The length of the phosphate chain and the concentration of the adenine nucleotide did not modulate the volume imbibed by the prediuresing individuals (Fig. 3b). However, a significant dose-dependent effect on the average prediuresed volume was generally observed within ligand concentrations, with no variation between ligands (Fig. 3c).

## Discussion

The ultimate decision of haematophagous insects to imbibe a blood meal is predominantly determined though the detection of blood-derived adenine nucleotides [1, 4, 8, 13–15]. In the present study, we found that the sensitivity for adenine nucleotides differed between *Ae. aegypti* and *An. gambiae*, while the specificity for these phagostimulants remained consistent, indicating a species-dependent tuning of the chemosensory neurons responding to adenine nucleotides. Moreover, *An. gambiae* detected the carbonate buffer in the absence of adenine nucleotides, although at a lower level than previously reported [8], suggesting that *An. gambiae* encodes blood phagostimulants in a manner that differs from *Ae. aegypti*. Both species displayed a dose-dependent bimodal feeding pattern on adenine nucleotide-containing meals, in which individuals either tasted or engorged, expanding the accepted dogma of all-or-none feeding response by haematophagous arthropods [9–11] to include an initial sampling phase. Taken together, the results of this comparative study expand our understanding of how mosquitoes differentially assess the constituents of the blood meal and provide a basis for further molecular and physiological studies.

Both *Ae. aegypti* and *An. gambiae* displayed a similar specificity for the adenine nucleotides, while *An. gambiae* was less sensitive to these phagostimulants than *Ae. aegypti*. The specificity of the gorging response of *Ae. aegypti* reflected previous descriptions, with the length of the phosphate chain correlated with the generation of a higher proportion of mosquitoes engorging [4, 13]. This selectivity is not reflected in the response of the apical labral gustatory receptor neurons [28], which raises the question of the mechanism by which behavioural specificity to adenine nucleotides is generated in *Ae. aegypti*. The current model for blood constituent detection stipulates polymodal taste integration using four chemosensory neurons denoted for their sensitivities, namely ATP, carbonate, saline and integrator, which detects a mix of NaHCO_3_, NaCl and glucose [29]. The integration of the signals from these channels may explain why saline and carbonate do not induce feeding in *Ae. aegypti* [13] and why saline together with ATP is a weak phagostimulant, whereas the combination of all three blood constituents produces a high proportion of engorgement at low concentrations of ATP [4, 13]. That the response of *An. gambiae* was dependent on the carbonate buffer indicates that the neuronal mechanism encoding behavioural specificity for adenine nucleotides is different in this species and thus requires further investigation. Of note, the differential carbonate-buffered saline response demonstrated in this study recapitulates that which was observed between the few culicines and anophelines studied previously [4, 8, 13, 30], indicating the possibility of lineage-specific polymodal taste integration.

The high sensitivity to ATP of *Ae. aegypti* and *An. gambiae* supports the dominance of ATP as a blood-feeding stimulant in an array of haematophagous species [3, 7, 10, 30–34], which has been postulated to arise via convergent evolution [35]. ATP is a ubiquitous intracellular metabolic energy currency released in the extracellular matrix upon rupture of epidermal and red blood cells, making it readily accessible to the sensory, piercing mouthparts of a haematophagous arthropod, placing ATP as a reliable signal for a fresh blood meal [36, 37]. However, species-specific variation in the preference for adenine nucleotide type [1] suggests structural differences in the putative purinoceptor, which may affect the affinity of cognate ligands, and subsequent feeding rate and volumes imbibed. The behavioural findings in this study suggest that such putative purinoceptors may be involved in regulating the reflexive drive to engorge, but also in an initial tasting phase of feeding. This result further advances the current understanding of the all-or-none blood-feeding hypothesis.

A generally accepted hypothesis is that haematophagous arthropods, regardless of the type of adenine nucleotide, feed to engorgement once stimulated, i.e. an all-or-none feeding response [9–11]. This study furthermore demonstrates that feeding on adenine nucleotides by both *Ae. aegypti* and *An. gambiae* mosquitoes follows a bimodal feeding pattern, describing a two-phase feeding behaviour, tasting followed by gorging, if a dose-dependent threshold is reached. Whether this is restricted to facultative blood-feeders, including mosquitoes, or also includes obligate blood-feeders, such as *Rhodnius prolixus* [9], which, while feeding on a broader range of phosphate-containing compounds, exhibit common neural connectivity to the stomatogastric and central nervous systems [38, 39], requires further analysis.

The effect of blood-related phagostimulants on prediuresis, a physiological response observed in *An. gambiae* but not *Ae. aegypti* [23, 40], is largely unknown. Prediuresis occurs rapidly following the onset of blood-feeding and has been implicated in both thermoregulation [23] and the concentration of red blood cells in the midgut of select anophelines [40, 41]. As ATP provides a fast and reliable signal of a blood meal, this adenine nucleotide is likely well positioned to play a role in regulating prediuresis in *An. gambiae* via stomatogastric signalling. This study demonstrates that the proportion of prediuresing individuals follows a similar pattern to the proportion of engorged individuals, which is dependent on the dose and type of ligand, predominantly ATP, and not blood protein content [this study, [42]]. The findings presented in this study suggest that adenine nucleotides and their structure regulate the probability of prediuresis in *An. gambiae,* and not the amount engorged among the prediuresing individuals.

## Conclusions

While both *Ae. aegypti* and *An. gambiae* demonstrate a consistent specificity for adenine nucleotides, these species demonstrate variable sensitivity to these phagostimulants. Moreover, in the present study, a consistent proportion of *An. gambiae* engorged on the bicarbonate alone, suggesting a species-dependent tuning of the chemosensory system responding to blood phagostimulants. The all-or-none blood-feeding hypothesis has been extended to include a dose-dependent initial tasting phase in these facultative blood-feeders. Moreover, the mechanism proposed for regulating prediuresis has been extended to include the adenine nucleotide detection in *An. gambiae*. As a whole, this study increases our understanding of how mosquitoes differentially encode and respond to blood phagostimulants and provides a basis for future physiological and molecular studies.

## Supplementary Information

Additional File 1: Figure S1. Feeding patterns in* Aedes aegypti* and *Anopheles gambiae* on adenine nucleotides.** a**,** b** The volumes imbibed among tasters (T) and engorgers (E) of* Ae. aegypti* (**a**) and* An. gambiae* (**b**) (N = 9, n = 90 for *Ae. aegypti* and N = 5, n = 50 for *An. gambiae*).** c** The proportions of* An. gambiae* engorged in the prediuresis experiment (*Nn* *N* = 5, *n* = 50 for* An. gambiae*). Comparisons between tasters and engorgers were made separately within the groups in** a** and** b**, corresponding to the statistical letters (a) and (a″), were performed using a two-way ANOVA (*P* < 0.05). Different letters indicate significant differences as determined by pairwise post-hoc tests. The error bars indicate the standard error of the proportion of engorged* An. gambiae* (*N* = 5, *n* = 50). ns, Non-significant.

Additional File 2: Figure S2. The effect of bovine serum albumin on prediuresis in *Anopheles gambiae*.** a**,** b** The proportion of engorged (**a**) and prediuresing individuals (**b**).** c** The volume engorged among prediuresing individuals.** d** The average volume prediuresed. The error bars indicate the standard error of the proportion and the volumes engorged and prediuresed (*N* = 5, *n* = 50). Different letters indicate significant differences as determined by pairwise post-hoc tests, following a one-way analysis of variance (*P* < 0.05) statistical test. ns, Non-significant.

## Data Availability

All data generated or analysed during this study are included in this published article and its supplementary information files. Data are provided within the manuscript or supplementary information files.

## Abbreviations

ADP: Adenosine diphosphate
AMP: Adenosine monophosphate
ATP: Adenosine triphosphate
cAMP: Cyclic adenosine monophosphate
