# Supplementary figures and images for: Differential sensitivity and specificity of Aedes aegypti and Anopheles gambiae to adenine nucleotide phagostimulants—an all-or-none response?
Source: Parasit Vectors. 2024 Nov 4;17:450. doi: 10.1186/s13071-024-06482-4 (PMC11536708; doi:10.1186/s13071-024-06482-4)

a)

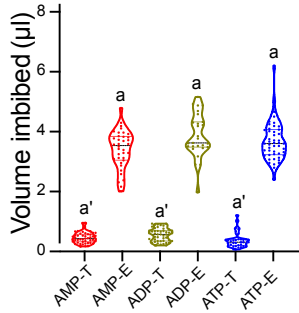

b)

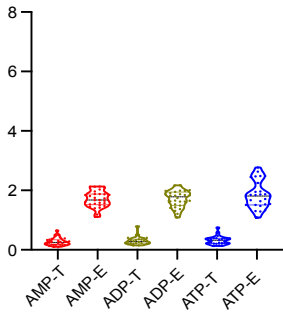

Ligands

c)

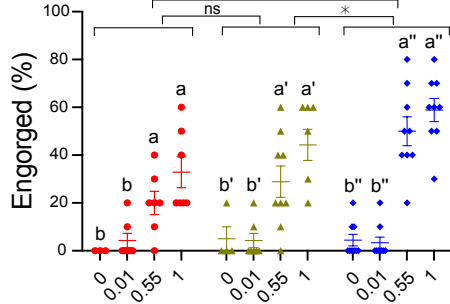

Concentration (mM)

Supplement: Supplementary file 1 — Additional File 1: Figure S1. Feeding patterns in Aedes aegypti and Anopheles gambiae on adenine nucleotides. a, b The volumes imbibed among tasters (T) and engorgers (E) of Ae. aegypti (a) and An. gambiae (b) (N = 9, n = 90 for Ae. aegypti and N = 5, n = 50 for An. gambiae). c The proportions of An. gambiae engorged in the prediuresis experiment (Nn N = 5, n = 50 for An. gambiae). Comparisons between tasters and engorgers were made separately within the groups in a and b, corresponding to the statistical letters (a) and (a″), were performed using a two-way ANOVA (P < 0.05). Different letters indicate significant differences as determined by pairwise post-hoc tests. The error bars indicate the standard error of the proportion of engorged An. gambiae (N = 5, n = 50). ns, Non-significant. [file 13071_2024_6482_MOESM1_ESM.pdf]

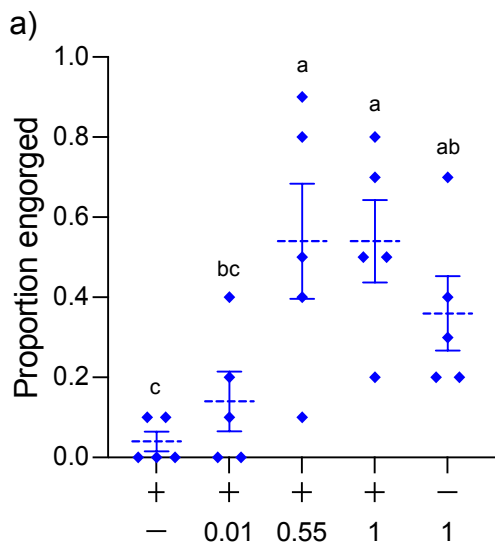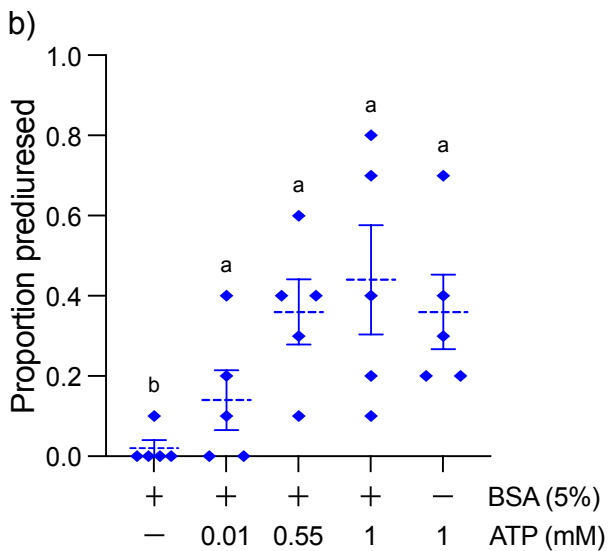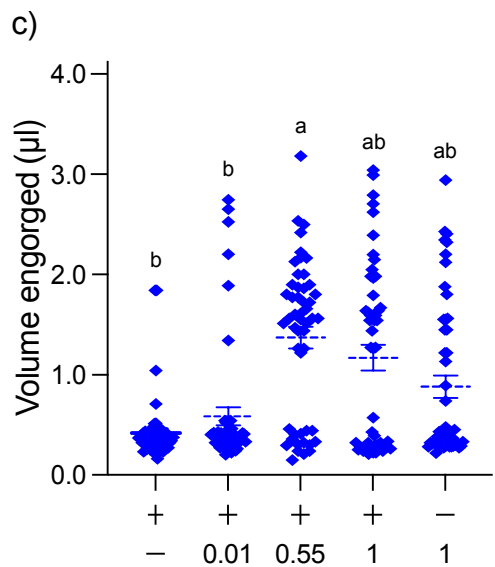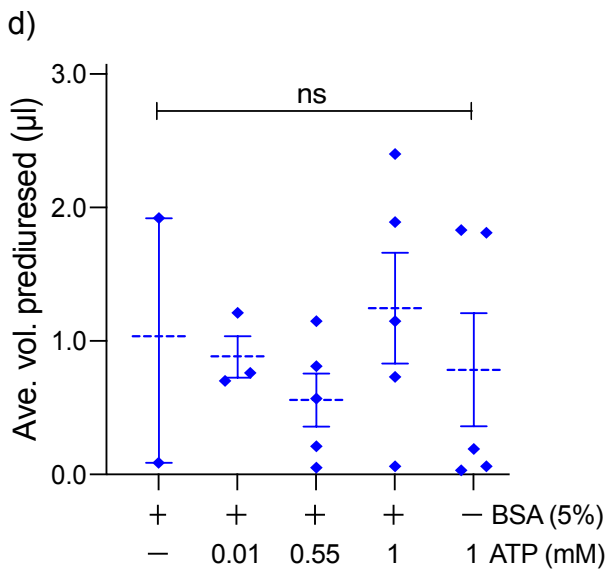

Diet concentrations

Supplement: Supplementary file 2 — Additional File 2: Figure S2. The effect of bovine serum albumin on prediuresis in Anopheles gambiae. a, b The proportion of engorged (a) and prediuresing individuals (b). c The volume engorged among prediuresing individuals. d The average volume prediuresed. The error bars indicate the standard error of the proportion and the volumes engorged and prediuresed (N = 5, n = 50). Different letters indicate significant differences as determined by pairwise post-hoc tests, following a one-way analysis of variance (P < 0.05) statistical test. ns, Non-significant. [file 13071_2024_6482_MOESM2_ESM.pdf]
